# DPSCs treated by TGF-β1 regulate angiogenic sprouting of three-dimensionally co-cultured HUVECs and DPSCs through VEGF-Ang-Tie2 signaling

**DOI:** 10.1186/s13287-021-02349-y

**Published:** 2021-05-10

**Authors:** Yuchen Zhang, Junqing Liu, Ting Zou, Yubingqing Qi, Baicheng Yi, Waruna Lakmal Dissanayaka, Chengfei Zhang

**Affiliations:** 1 Restorative Dental Sciences, Faculty of Dentistry, The University of Hong Kong, Hong Kong, China; 2 Applied Oral Sciences & Community Dental Care, Faculty of Dentistry, The University of Hong Kong, Hong Kong, China

**Keywords:** Dental pulp stem cells, Smooth muscle cells, Angiogenesis, Ang1/Tie2 signaling, Vessel stability

## Abstract

**Background:**

Maintaining the stability and maturation of blood vessels is of paramount importance for the vessels to carry out their physiological function. Smooth muscle cells (SMCs), pericytes, and mesenchymal stem cells (MSCs) are involved in the maturation process of the newly formed vessels. The aim of this study was to investigate whether transforming growth factor beta 1 (TGF-β1) treatment could enhance pericyte-like properties of dental pulp stem cells (DPSCs) and how TGF-β1-treated DPSCs for 7 days (T-DPSCs) stabilize the newly formed blood vessels.

**Methods:**

We utilized TGF-β1 to treat DPSCs for 1, 3, 5, and 7 days. Western blotting and immunofluorescence were used to analyze the expression of SMC markers. Functional contraction assay was conducted to assess the contractility of T-DPSCs. The effects of T-DPSC-conditioned media (T-DPSC-CM) on human umbilical vein endothelial cell (HUVEC) proliferation and migration were examined by MTT, wound healing, and trans-well migration assay. Most importantly, in vitro 3D co-culture spheroidal sprouting assay was used to investigate the regulating role of vascular endothelial growth factor (VEGF)-angiopoietin (Ang)-Tie2 signaling on angiogenic sprouting in 3D co-cultured spheroids of HUVECs and T-DPSCs. Angiopoietin 2 (Ang2) and VEGF were used to treat the co-cultured spheroids to explore their roles in angiogenic sprouting. Inhibitors for Tie2 and VEGFR2 were used to block Ang1/Tie2 and VFGF/VEGFR2 signaling.

**Results:**

Western blotting and immunofluorescence showed that the expression of SMC-specific markers (α-SMA and SM22α) were significantly increased after treatment with TGF-β1. Contractility of T-DPSCs was greater compared with that of DPSCs. T-DPSC-CM inhibited HUVEC migration. In vitro sprouting assay demonstrated that T-DPSCs enclosed HUVECs, resembling pericyte-like cells. Compared to co-culture with DPSCs, a smaller number of HUVEC sprouting was observed when co-cultured with T-DPSCs. VEGF and Ang2 co-stimulation significantly enhanced sprouting in HUVEC and T-DPSC co-culture spheroids, whereas VEGF or Ang2 alone exerted insignificant effects on HUVEC sprouting. Blocking Tie2 signaling reversed the sprouting inhibition by T-DPSCs, while blocking VEGF receptor (VEGFR) signaling boosted the sprouting inhibition by T-DPSCs.

**Conclusions:**

This study revealed that TGF-β1 can induce DPSC differentiation into functional pericyte-like cells. T-DPSCs maintain vessel stability through Ang1/Tie2 and VEGF/VEGFR2 signaling.

**Supplementary Information:**

The online version contains supplementary material available at 10.1186/s13287-021-02349-y.

## Background

Dental pulp stem cells (DPSCs), which generally reside within the sub-odontoblast layer, pulpal vasculature, and central pulp region, are responsible for odontoblast formation and vascular cell replenishment, respectively [1, 2]. DPSCs possess all the properties of mesenchymal stem cells (MSCs) and demonstrate the ability to differentiate into various primary cell phenotypes, such as neuronal, endothelial, and smooth muscle cells (SMCs) when triggered by microenvironmental factors such as signaling molecules and growth factors [2]. A previous study investigated the angiogenic and pericyte function of DPSCs and showed that DPSCs promote angiogenesis by secreting vascular endothelial growth factor (VEGF) and acting as pericyte-like cells to stabilize vessels [3]. Likewise, our recent studies showed that delayed administration of DPSCs could stabilize the preexisting vessel-like structures formed by human umbilical vein endothelial cells (HUVECs) and increase the longevity of them [4]. Further, in vitro Matrigel assay and in vivo studies corroborated that DPSCs often closely associate with vessels and adopt the pericyte location and function [3, 5]. It was reported that TGF-β1/ALK5 is one of the signaling pathways involved in regulating the capacity of DPSCs to differentiate into mural cells, which express α-smooth muscle actin (α-SMA), calponin, and platelet-derived growth factor receptors (PDGFR) [5]. On the other hand, the crosstalk between endothelial cells (ECs) and DPSCs via EphrinB2/EphB4 signaling regulates angiogenic sprouting [6], while signaling events triggered by nearby vascular ECs within the perivascular niche is a prerequisite for the maintenance of stemness of DPSCs [7].

Angiogenesis, the process of sprouting and maturation of capillaries, is regulated by multiple factors, signaling pathways, and cellular interactions [8, 9]. It has multiple steps: EC activation and pericyte separation from the endothelium, EC sprouting guided by growth factor gradients, anastomosis of immature endothelial sprouts, and maturation of vessels through recruitment of mural cells [9]. In the maturation step, the assembled endothelial cells deposit a new basement membrane and secrete pro-maturation factors, such as platelet-derived growth factor (PDGF), which promotes the recruitment of pericytes or SMCs that closely associate with the nascent vessels and stabilize them [8–10]. Similarly, dental stem cells, when interact with HUVECs, recapitulate the process of angiogenesis. DPSCs increase early vascular network formation by promoting the migration of HUVECs via VEGF secretion, whereas the coordinated crosstalk between HUVECs and DPSCs plays crucial roles in fine-tuning angiogenic sprouting and vessel stabilization [11]. It was found that VEGF secretion is precisely orchestrated parallel to the different stages of the angiogenic process [11, 12]. Other MSCs, such as bone marrow mesenchymal stem cells (BMMSCs) and adipose stem cells (ASCs), interact with ECs similarly, shifting their paracrine activity from being pro-angiogenic to angiostatic and phenotypes from stem cells to mural cells following the developmental progress of the vessel formation [13–16].

Angiopoietin 1 (Ang1), the ligand of Tie2, can induce Tie2 phosphorylation in ECs and is responsible for blood vessel stabilization [17, 18]. Activation of Ang1/Tie2 signaling can maintain the quiescent EC phenotype and strengthen the interactions between pericytes/SMCs and ECs [18, 19]. In addition, ECs via Ang1/Tie2 activation recruit peripheral cells and vascular SMCs to stabilize and maturate the newly formed blood vessels [20]. Angiopoietin 2 (Ang2) has an opposing function and competes for the same Tie2 receptor with Ang1 by destabilizing blood vessels [21, 22]. Since DPSCs interacting with HUVECs recapitulate the process of angiogenesis, we hypothesized that similar signaling pathways may regulate this process, in particular, activation of Ang1/Tie2 signaling may stabilize blood vessels within 3-dimensional (3D) co-cultured HUVECs and DPSCs.

Transforming growth factor beta 1 (TGF-β1) protein is the most effective soluble growth factor regularly utilized for inducing stem cell differentiation into SMCs [5, 16, 23]. TGF-β1/Smad signaling pathway is critical for differentiation of mesenchymal stem cell/progenitors into SMCs [24]. Similarly, dental stem cells have been successfully induced into functional SMCs for vascular engineering by TGF-β1 treatment and this course could be regulated through the ALK5 signaling pathway [5, 24]. Therefore, in order to explore the relevant signaling pathways between pericyte-like DPSCs and ECs, T-DPSCs were utilized in this study to mimic the pericyte function.

In this study, a well-established in vitro 3D co-culture collagen gel model was employed to mimic multiple stages of angiogenesis [25]. We aimed to investigate (1) the dynamic expression of growth factors in the process of DPSC differentiation into functional pericyte-like cells, (2) whether T-DPSCs function like pericytes stabilizing the newly formed blood vessels, and (3) whether VEGF-Ang-Tie2 signaling is involved in the crosstalk between pericyte-like DPSCs and ECs.

## Methods

### Cell culture

DPSCs were purchased from Lonza (Basel, Switzerland) and cultured in α-modified Eagle’s medium (α-MEM) (Thermo Fisher Scientific, Waltham, MA, USA) supplemented with 10% (v/v) fetal bovine serum (FBS) (Thermo Fisher Scientific) and 1% (v/v) P/S (penicillin/streptomycin) (Sigma-Aldrich, St. Louis, MO, USA). The stemness of the obtained cells was characterized by flow cytometry and the multiple differentiation capacity.

HUVECs were purchased from Lonza (Basel, Switzerland) and cultured in a fully supplemented endothelial growth medium (EGM-2, Lonza, Walkersville, MD, USA). Human brain vascular pericytes (HBVPs) and SMCs were purchased from ScienCell (ScienCell, Carlsbad, CA, USA) and cultured in a pericyte medium (PM, ScienCell) and a smooth muscle cell medium (SMCM, ScienCell), respectively. All cells were cultured in an incubator under 5% CO_2_ at 37 °C.

### Flow cytometry

For flow cytometry, 5×10^5^ DPSCs were collected and resuspended in 100 μL PBS. Primary antibody was added and incubated for 60 min at 4°C in the dark. The antibodies were as follows: mouse anti-human fluorescein isothiocyanate (FITC)-labeled monoclonal anti-CD90 (#561254), mouse anti-human FITC-labeled monoclonal anti-CD73 (#555595), mouse anti-human phyeoerythrin (PE)-labeled monoclonal anti-CD45 (#560975), and mouse anti-human PE-labeled monoclonal antiCD34 (#560941). FITC-labeled mouse immunoglobulin G (IgG) (#554679) and PE-labeled mouse IgG (#555574) were used as negative control antibodies. The antibodies were all purchased from BD Biosciences (San Jose, CA, USA). Data of 20,000 stained cells were collected and analyzed using a FACSCalibur instrument and FACSCalibur software (BD Biosciences).

### Osteogenic differentiation

For osteogenic differentiation, DPSCs were seeded into a 6-well plate at 2×10^3^ cells/cm^2^ and cultured in an osteogenic induction medium which consisted of 10% FBS (Thermo Fisher Scientific), 10 mM β-glycerolphosphate (Sigma-Aldrich), 10 nM dexamethasone (Sigma-Aldrich), and 50 mg/L L-ascorbic acid (Sigma-Aldrich). The medium was replaced every 3 days. After 21 days of induction, cells were fixed with 4% paraformaldehyde (PFA) for 30 min and stained with 2% Alizarin red (Sigma-Aldrich) for 10 min. Then, the cells were washed three times with distilled water and taken pictures by the microscope (Nikon, Tokyo, Japan).

### Neurogenic differentiation

For neurogenic differentiation, DPSCs at passages 3–5 were seeded into a 6-well plate at 1×10^4^ cells/cm^2^ and cultured in a neurogenic induction medium for 7 days. The induction medium contained DMEM/F12: neurobasal [1:1] supplemented with 0.5% [v/v] N2, 1% [v/v] B27, 100 μM cyclic adenosine monophosphate (cAMP), 20 ng/mL basic fibroblast growth factor (bFGF), and 1% penicillin/streptomycin. The reagents were all purchased from Thermo Fisher Scientific. The induction medium was replaced every 3 days. Then, the immunofluorescence analysis was performed as below immunofluorescence method.

### Chondrogenic differentiation

For chondrogenic differentiation, 4×10^5^ DPSCs were placed into a 15-mL polypropylene tube and centrifuged for 4 min at 250×*g*. The cell pellets were resuspended in the chondrogenic induction medium (Cyagen Biosciences, Guangzhou, China). The induction medium was replaced every 3 days. After 28 days of induction, pellets were formalin fixed and paraffin embedded. Thin sections were stained with Alcian blue (Cyagen Biosciences) to visualize proteoglycans in the extracellular matrix.

### Adipogenic differentiation

For adipogenic differentiation, DPSCs were seeded at a density of 2×10^4^ cells/cm^2^ in a 6-well plate until the cell reached 100% confluence. Then, the medium was changed to adipogenic induction medium (Cyagen Biosciences) for 3 days and subsequently maintained in adipogenic maintenance medium (Cyagen Biosciences) for 24 h. After 28 days of induction, cells were fixed with 4% PFA for 30 min and stained with Oil red O solution (Sigma-Aldrich) for 30 min to visualize lipid vacuoles.

### RT-qPCR

Real-time quantitative PCR polymerase chain reaction (RT-qPCR) was performed as described previously [12]. The primers used in this study are listed in Table 1. The glyceraldehyde 3-phosphate dehydrogenase (GAPDH) was used as an internal control, and the relative expression of interested genes was measured by the 2^−ΔΔCt^ method.

**Table 1:** RT-qPCR primer sequences

Primers	Sequences	References
GAPDH	Forward: TGCACCACCAACTGCTTAGC Reverse: GGCATGGACTGTGGTCATGAG	NM_001256799
α-SMA	Forward: CCGACCGAATGCAGAAGGA Reverse: ACAGAGTATTTGCGCTCCGAA	NM_001141945
SM22α	Forward: AGTGCAGTCCAAAATCGAGAAG Reverse: CTTGCTCAGAATCACGCCAT	NM_001001522

### Western blotting

Western blotting was performed to analyze the protein expression as previously described [6]. Briefly, equal amounts of the protein extracts were loaded and separated by SDS-PAGE and transferred to PVDF membranes. Then, the PVDF membranes were blocked in 5% milk at room temperature for 1 h and incubated with the following primary antibodies (dilution 1:1000) overnight at 4 °C: anti-SM22α (#ab14106), anti-Ang1 (#ab183701), anti-VEGF-A (#ab46154), and anti-Tie2 (#ab24859), which were purchased from Abcam company (Abcam, Cambridge, UK); anti-α-SMA (#19245S), anti-p-VEGFR2 (Tyr1175) (#2478S), anti-VEGFR2 (#2479S), anti-p-Smad2 (Ser465/467) (#3108T), anti-p-Smad3 (#Ser423/425) (#9520T), anti-Smad2/3 (#8685T), VE-Cadherin (#2500S), and anti-GAPDH (#2118S) which were purchased from Cell Signaling Technology (CST, MA, USA); and anti-p-Tie2 (Tyr992) (#AF2424) which was purchased from Affinity Biosciences (Cincinnati, OH, USA). After washing three times, the membranes were incubated with a secondary antibody and then visualized by using Pierce ECL western blotting substrate (Thermo Fisher Scientific). Quantification was performed with ImageJ software (Bethesda, MD, USA)

### Immunofluorescence

Cells were fixed with 4% (w/v) cold PFA for 15 min and washed with PBS for three times. Then, cells were permeabilized using 0.3% (v/v) Triton X-100 in PBS with 5% FBS for 1 h. Primary antibodies against α-SMA (#19245S, CST), SM22α (#ab14106, Abcam), and beta III Tubulin (Tuj1) (#ab78078, Abcam) were used. Alexa Fluor 488-conjugated goat anti-mouse antibody and Alexa Fluor 647®-conjugated goat anti-rabbit antibody were used as the secondary antibody and nuclei were stained with DAPI (Thermo Fisher Scientific). Images were captured by a fluorescence microscope (Nikon).

### Functional contraction assay

The contraction assay was performed as previously described [26]. Briefly, DPSCs and T-DPSCs were collected, and the cell suspension was prepared at the density of 2×10^5^ cells/mL. The collagen mix was prepared on ice using Collagen type I (Thermo Fisher Scientific), Medium 199 (Thermo Fisher Scientific), and NaOH (1 M) in an 8:1:1 ratio. The collagen solution (0.6 mL) and cell suspension (1.2 mL) were mixed thoroughly by pipette and 0.5 mL of the mixture was immediately transferred to a well of 24-well plate. For polymerization, gels were incubated at 37°C for 30 min and then 100 μL α-MEM was added to each well. After 48 h, the diameter of the gel was measured with ImageJ software (Bethesda).

### Conditioned media (CM)

DPSCs were treated with TGF-β1 (10 ng/mL) for 7 days. Then, the untreated DPSCs and T-DPSCs were cultured until 80% confluence in α-MEM. Then, the medium was changed to a serum-free medium. After 48 h, conditioned media were collected, centrifuged at 1000 rpm, and filtered with a 0.2-μm filter to remove cell debris.

### MTT assay

To determine the effect of CM from T-DPSC (T-DPSC-CM) on proliferation of endothelial cells, HUVECs were seeded on a 96-well plate at a density of 3000 cells per well. After 24 h, the mixtures of DPSC-CM or T-DPSC-CM with EGM2 (1:1) were added to the corresponding wells. Cell numbers were determined by MTT (3-(4,5-dimethylthiazol-2-yl)-2,5-diphenyltetrazolium bromide) (Molecular Probe, OR, USA) at different time points of 0, 24, 48, and 72 h. Briefly, the medium was replaced with a 100-μL fresh medium and 10 μL 12 mM MTT solution was added to each well. After incubation for 4 h at 37 °C, the medium was removed leaving only 25 μL in the well. Then, 50 μL DMSO was added to each well and mixed thoroughly. After incubation for 10 min at 37 °C, the absorbance of each well at 540 nm was measured with a SpectraMax®M2microplate reader (Molecular Devices, CA, USA).

### Wound healing assay

HUVECs were seeded on a 6-well plate and cultured until 80% confluence. A constant diameter strip was made using 200-μL pipette tip across the center of the well. After scratching, the cells were washed with PBS to remove cell debris. Then, 2 mL DPSC-CM or T-DPSC-CM was added to each well and cultured for 24 h. Photographs were taken at 0 h and 24 h under a microscope (Nikon). Six randomly selected fields of the wound were marked, and the width of the wound was measured with ImageJ software (Bethesda).

### Trans-well migration assay

HUVECs were collected and suspended in serum-free medium and plated in the top chamber (24-well insert; pore size, 8 mm; Corning, NY, USA) at a density of 1 × 10^4^ cells/0.2 mL. Next, 600 μL of DPSC-CM or T-DPSC-CM was added to the lower compartment of the corresponding wells. After 24 h, cells that did not migrate through pores on the upper surface of the membrane were removed by a cotton swab. Cells that migrated through the pores were fixed with 4% PFA for 15 min and stained with 0.1% (w/v) crystal violet (Sigma) for 15 min. Five randomly selected fields of each well were selected under the inverted microscope (Nikon) and cell number was counted by ImageJ (Bethesda).

### In vitro 3D sprouting assay

In order to investigate the function of T-DPSCs in endothelial sprouting, the 3D spheroid sprouting assay was performed as described previously [25]. Briefly, HUVECs and DPSCs or T-DPSCs were mixed 1:1 at a density of 4×10^4^ cells/mL in α-MEM containing 20% Methocel (Sigma). Twenty-five-microliter cell suspension drops were pipetted on non-adherent plastic plates. Subsequently, plates were turned upside-down and incubated for 24 h at 37 °C. The spheroids were collected and centrifuged at 200*g* for 5 min and resuspended in Methocel supplemented with 20% FBS. The collagen mix was prepared on ice using Collagen type I (Thermo Fisher Scientific), Medium 199, and NaOH (1 M) in an 8:1:1 ratio. The collagen solution was mixed with the spheroid solution in a 1:1 ratio and the 1-mL mixture was transferred to a 24-well plate. For polymerization, gels were incubated at 37 °C for 30 min. The assay was stopped after 24 h of incubation at 37 °C by adding 1 mL 4% PFA per well. Images of ten spheroids per gel were taken using a microscope (Nikon) and cumulative sprouting length was measured with ImageJ software (Bethesda).

### HUVEC and T-DPSC co-culture

HUVECs and T-DPSCs were collected and suspended in EGM2 and α-MEM medium, respectively. 4×10^5^ HUVECs and 4×10^5^ T-DPSCs were co-cultured in 6-well plates with or without Ang2. After 24 h, cells were lysed for western blot analysis.

### Growth factors and inhibitors

Recombinant human VEGF165 (Peprotech, Cranbury, USA) was reconstituted in PBS containing 0.1% BSA and the working concentration is 20 ng/mL. Recombinant human Ang1 and Ang2 (R&D system, MN, USA) were reconstituted in 0.9% NaCl containing 0.1% BSA and used at a concentration of 200 ng/mL and 1000 ng/mL, respectively. Tie2 kinase inhibitor (Cayman Chemical, MI, USA) was reconstituted in DMSO and used at a concentration of 5 μM. The inhibitor of VEGFR2, semaxinib (Med Chem Express, NJ, USA), was used at a concentration of 5 μM.

### Statistical analysis

All experiments were performed at least 3 times independently and all data were presented as mean ± standard deviation (SD). Student’s *t* test was performed between two groups and one-way ANOVA with a Tukey’s post hoc test was utilized in multiple comparisons for statistical analysis. *p* < 0.05 was considered statistically significant.

## Results

### Characterization of DPSCs

**Fig. 1:**
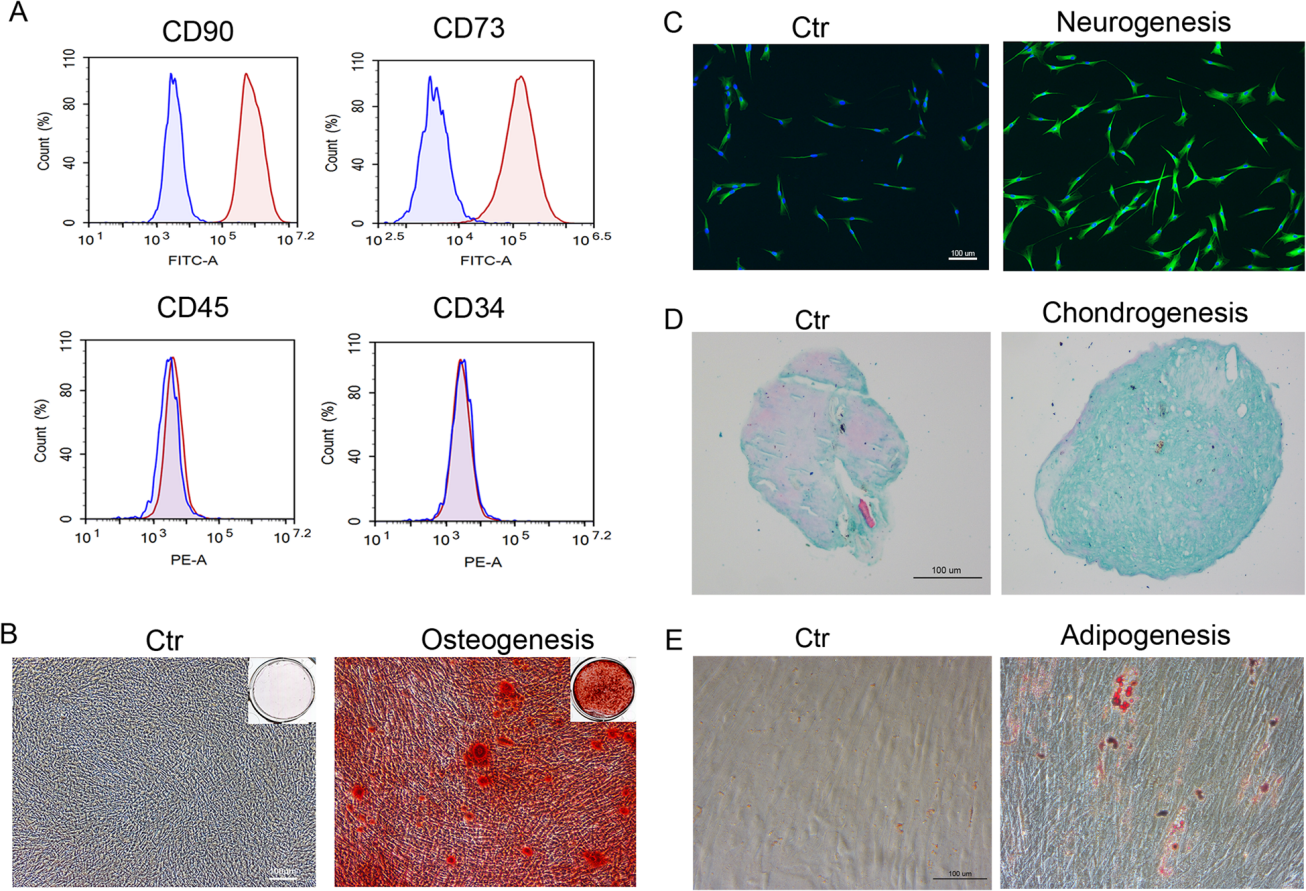
Characterization of DPSCs. **a** Specific marker analysis of DPSCs by flow cytometry. **b** Alizarin red staining after osteogenic induction for 21 days. **c** Immunofluorescence staining after neurogenic induction for 7 days (green: Tuj1, blue: DAPI). **d** Alcian blue staining after chondrogenic induction for 28 days. **e** Oil red O staining after adipogenic induction for 28 days

The obtained DPSCs were isolated from a wisdom tooth of an 18-year-old female. Through flow cytometry assessment, it was confirmed that the isolated DPSCs were positive for mesenchymal markers CD90 and CD73 while negative for hematopoietic markers CD45 and CD34 (Fig. 1a). Furthermore, DPSCs demonstrated multipotent differentiation capacity into osteogenic, neurogenic, chondrogenic, and adipogenic lineages (Fig. 1b–e).

### TGF-β1 induced DPSC differentiation into functional SMCs

**Fig. 2:**
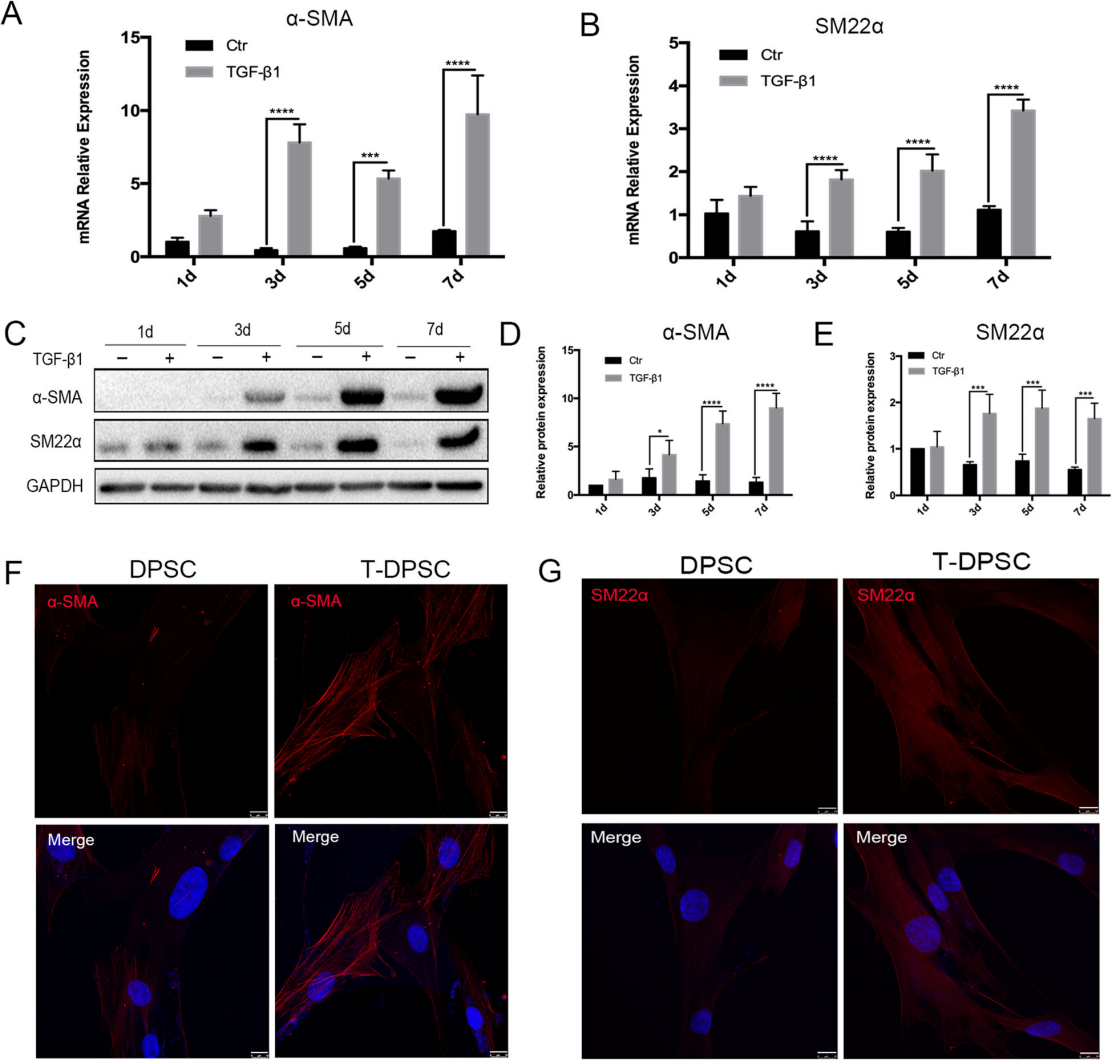
TGF-β1 induced the expression of α-SMA and SM22α in DPSCs. **a**, **b** RT-qPCR analysis of the relative mRNA expression of α-SMA and SM22α in DPSCs after treatment with TGF-β1 (10 ng/mL) for 1, 3, 5, and 7 days. **c**–**e** Western blot analysis of the expression of α-SMA and SM22α in DPSCs after treatment with TGF-β1 (10 ng/mL) for 1, 3, 5, and 7 days. **f**, **g** Representative immunofluorescence images of α-SMA (red) and SM22α (red) in DPSCs treated with TGF-β1 for 7 days. Scale bar represents 10 μm. Data are mean ± standard error for *n* = 3 replicates, *p < 0.05, ***p <0.001, ****p <0.0001

To verify the role of TGF-β1 in driving DPSC differentiation into functional SMCs, DPSCs were treated with TGF-β1 for different time points. As shown in Fig. 2, the expression of SMC-specific markers (α-SMA and SM22α) in DPSCs were significantly increased both at mRNA (Fig. 2a, b) and protein levels (Fig. 2c–e) after treatment with TGF-β1. Immunofluorescence corroborated the upregulated expression of α-SMA and SM22α in TGF-β1-treated DPSCs at 7 days. Consistent with the western blot results, the fluorescence intensity of α-SMA (Fig. 2f) and SM22α (Fig. 2g) was significantly higher in T-DPSCs than that in DPSCs.

**Fig. 3:**
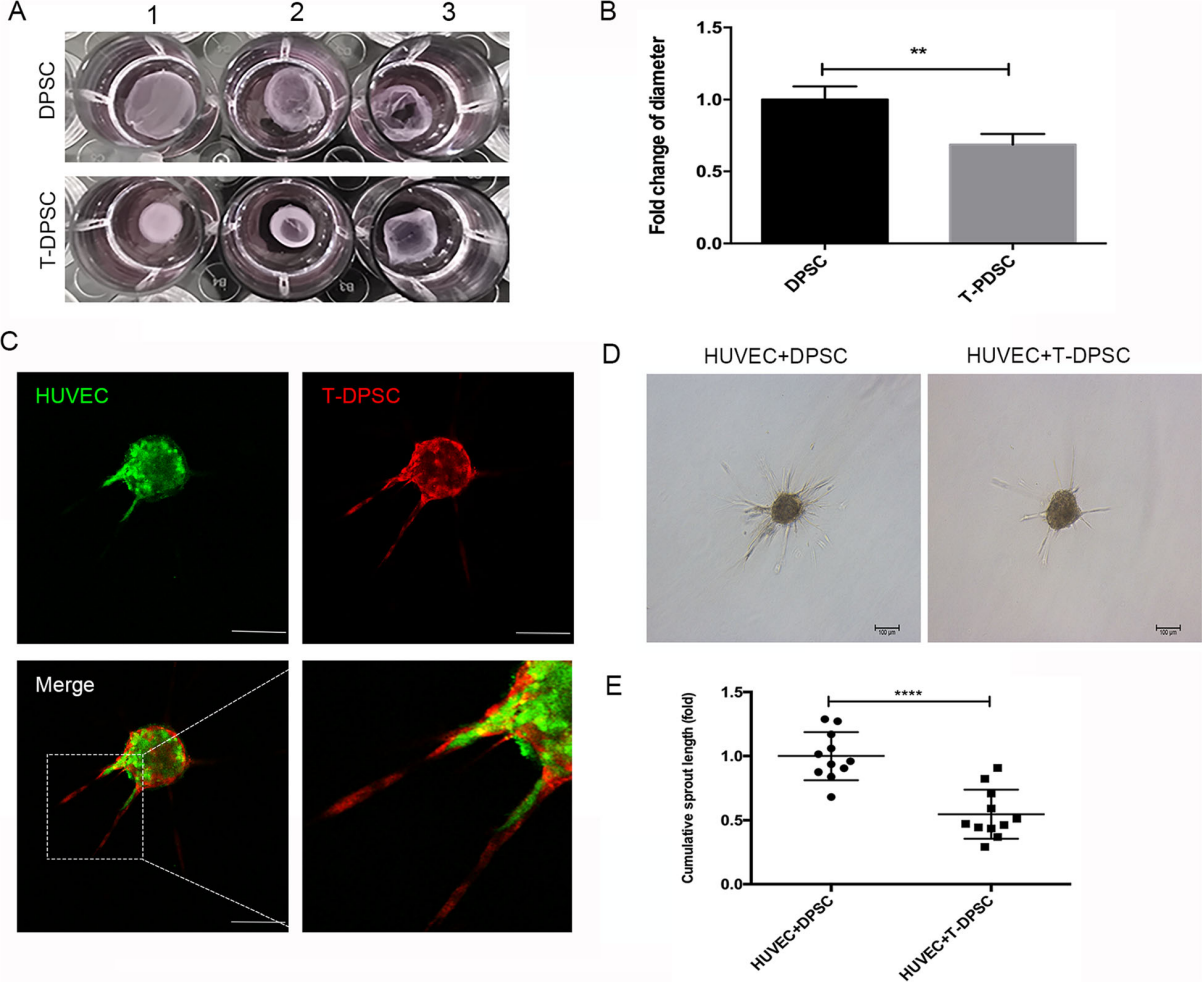
T-DPSCs exhibited similar functional properties as SMCs. **a** Contraction assay of the contractility of T-DPSCs in collagen gel. 1, 2, and 3 are three replicates. **b** The gel diameter was measured and analyzed using ImageJ software after T-DPSC seeding in the gels for 48 h. **c** Confocal laser microscopy images showed the location of HUVECs and T-DPSCs in sprouting structures. HUVECs and T-DPSCs were labeled with green and red cell tracker dyes, respectively. Scale bar represents 100 μm. **d**, **e** T-DPSCs significantly inhibited the sprouting of HUVECs in a 3D spheroid model. Data are mean ± standard error for *n* = 3 replicates, **p <0.01, ****p <0.0001

To investigate the contractile function of T-DPSCs, the contraction assay was performed. After 48 h, the diameter of the gel was measured, and the results revealed that the contractility of T-DPSCs was much stronger than that in DPSCs (Fig. 3a, b). To explore whether T-DPSCs affect the angiogenic sprouting of ECs in the 3D spheroids, co-culture spheroids of HUVECs with DPSCs or T-DPSCs were embedded in collagen gel. Fluorescent images showed that HUVECs were enclosed by T-DPSCs, which resembled pericyte-like cells (Fig. 3c). The EC sprouting was quantitated after 24 h and the results showed that T-DPSCs significantly inhibited EC sprouting in the 3D spheroidal co-culture model (Fig. 3d, e). Based on the above results, it could be concluded that TGF-β1 can induce DPSC differentiation into functional SMCs.

### T-DPSC-CM inhibited EC migration

**Fig. 4:**
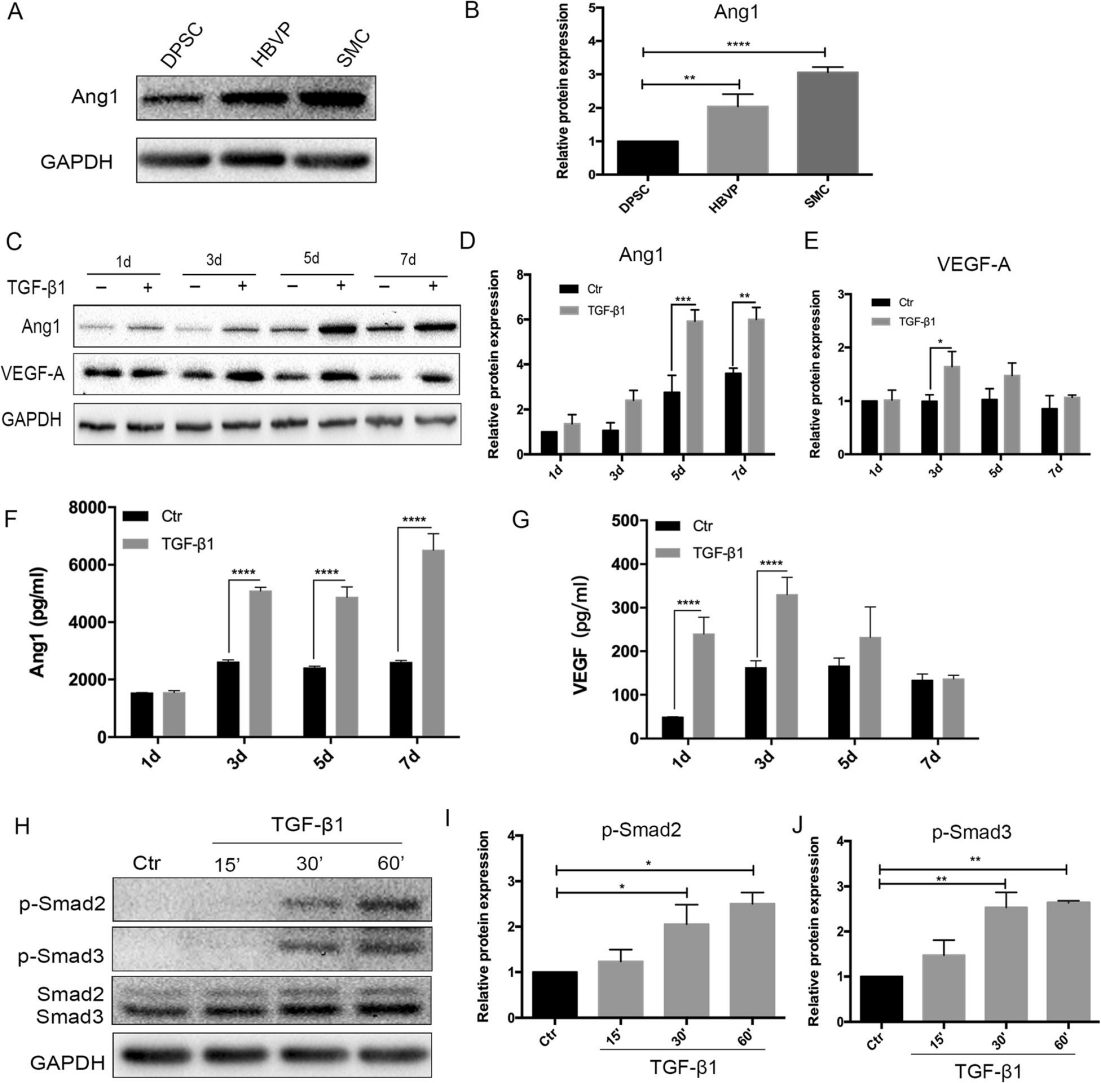
TGF-β1 regulated Ang1 and VEGF expression and secretion in DPSCs. **a**, **b** Western blot analysis of the expression of Ang1 in DPSCs, HBVPs, and SMCs. **c**–**e** Western blot analysis of the expression of Ang1 and VEGF in DPSCs treated with TGF-β1 (10 ng/mL) for 1, 3, 5, and 7 days. **f**, **g** ELISA analysis of the level of Ang1 and VEGF in conditioned media from DPSCs and T-DPSCs at 1, 3, 5, and 7 days. **h**–**j** Western blot analysis of the expression of p-Smad2 and p-Smad3 in DPSCs treated with TGF-β1 (10 ng/mL) at 15, 30, and 60 min. Data are mean ± standard error for *n* = 3 replicates, *p <0.05, **p <0.01, ***p <0.001, ****p <0.0001

To investigate whether T-DPSCs inhibit EC sprouting through Ang1/Tie2 and VEGF/VEGFR2 signaling, the expression and secretion of Ang1 and VEGF in T-DPSCs was analyzed. First, we verified that DPSCs expressed Ang1, which was much lower than that in HBVPs and SMCs (Fig. 4a, b). After treatment with TGF-β1 for 5 days and 7 days, Ang1 expression was significantly increased in T-DPSCs (Fig. 4c, d). The peak of VEGF expression was at 3 days after treatment with TGF-β1, and then the expression was downregulated gradually at 5 days and 7 days (Fig. 4c, e). Since both Ang1 and VEGF are secreted proteins, in order to play their roles, it is essential for them to be secreted from the cells to activate their corresponding receptors. Therefore, an ELISA assay was performed to detect the level of Ang1 and VEGF in DPSC-CM and T-DPSC-CM. The ELISA results showed that Ang1 concentration in T-DPSC-CM significantly increased at days 3, 5, and 7 (Fig. 4f). However, the VEGF level in T-DPSC-CM increased at 1 day and 3 days and then decreased gradually and reached the same level as that in DPSC-CM at 7 days (Fig. 4g). In order to investigate whether TGF-β1 through the downstream signaling Smad2 and Smad3 to trigger target gene transcription, such as α-SMA, SM22α, Ang1, and VEGF, the phosphorylation of Smad2 and Smad3 was assessed in DPSCs after treatment with TGF-β1 at different time points. Western blot results showed that the expression of p-Smad2 and p-Smad3 was significantly increased at 30 and 60 min after treatment with TGF-β1 (Fig. 4h–j).

**Fig. 5:**
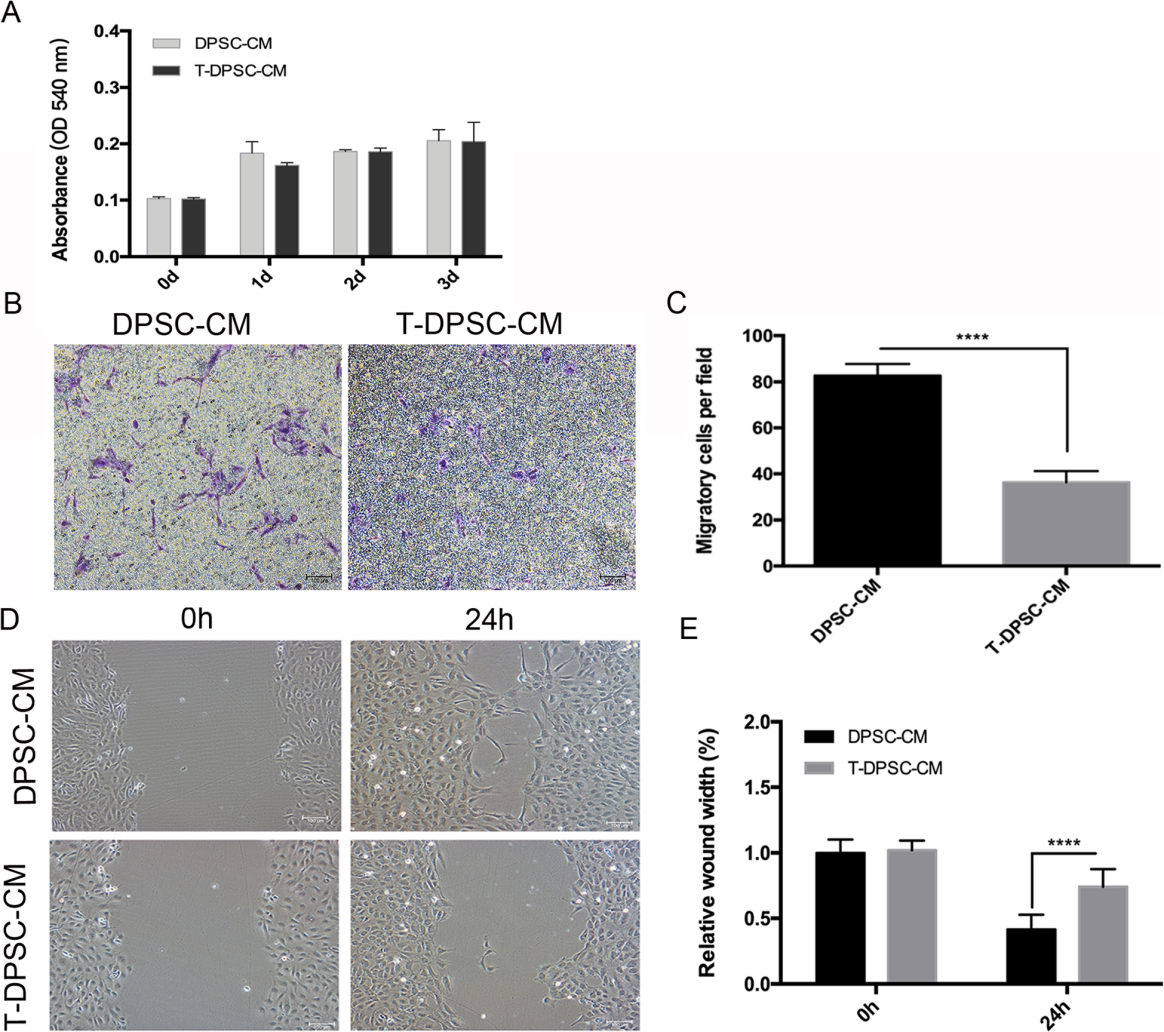
Conditioned media from T-DPSCs inhibited HUVEC migration. **a** HUVEC proliferation in DPSC-CM and T-DPSC-CM was determined by MTT assay. **b**, **c** Trans-well migration assay was performed to evaluate the effect of DPSC-CM and T-DPSC-CM on HUVEC migration ability. **d**, **e** HUVEC migration was determined by wound healing assay under DPSC-CM and T-DPSC-CM for 24 h. Data are mean ± standard error for *n* = 3 replicates, ****p <0.0001

To assess the effect of T-DPSC-CM on EC proliferation, an MTT assay was performed. The results indicated that T-DPSC-CM did not affect EC proliferation (Fig. 5a). The trans-well migration assay showed that a significantly lower number of ECs migrated to the bottom compartment in T-DPSC-CM than that in DPSC-CM (Fig. 5b, c). The results of the wound healing assay demonstrated that the cells migrated into the scratch area significantly slower under T-DPSC-CM than that in DPSC-CM (Fig. 5d, e).

**Fig. 6:**
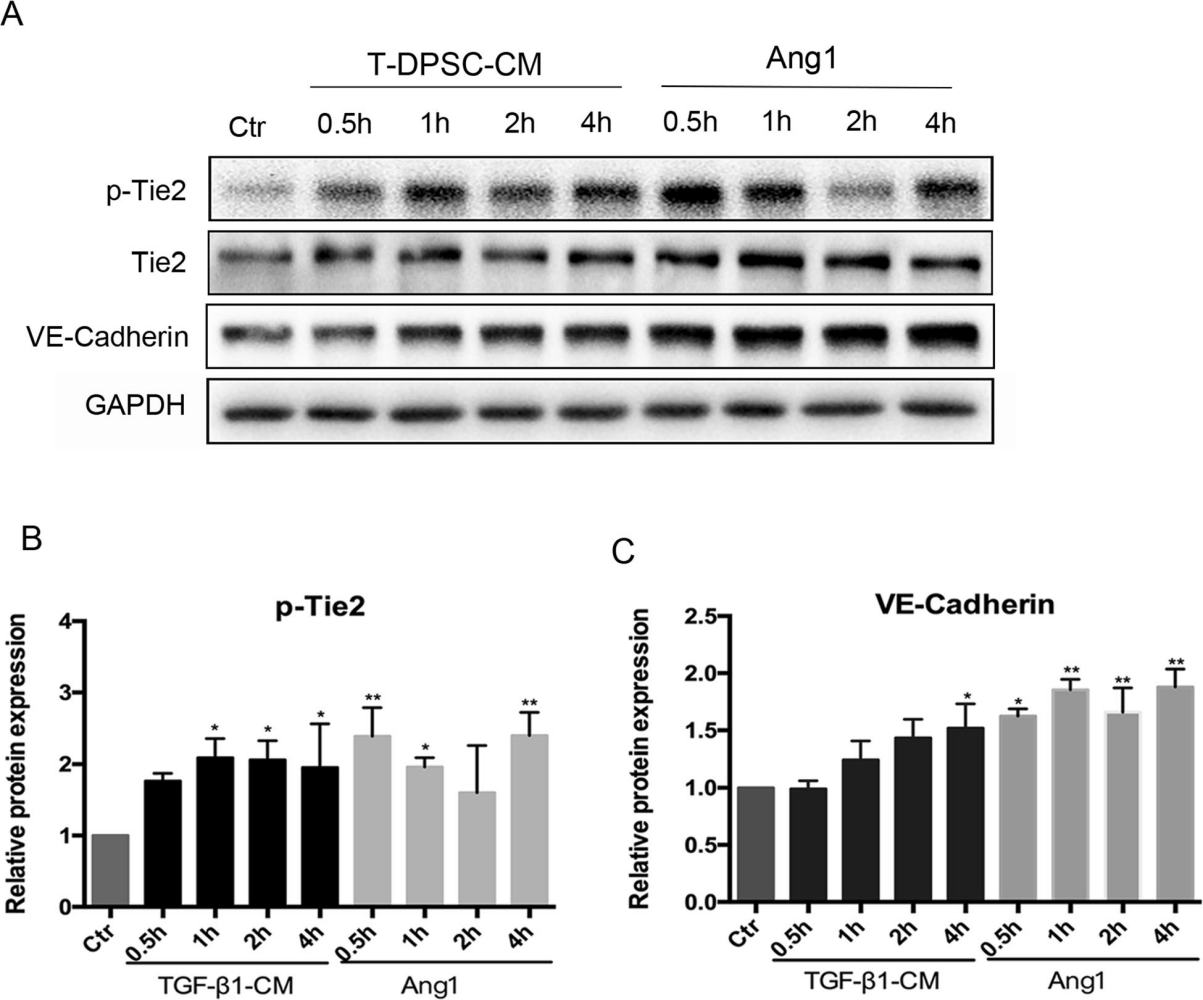
Conditioned media from T-DPSCs activated Tie2 signaling. **a**–**c** Western blot analysis of the expression of p-Tie2 and VE-Cadherin in HUVECs after treatment with T-DPSC-CM or Ang1 at different time points. Data are mean ± standard error for *n* = 3 replicates, *p <0.05, **p <0.01

As Ang1 concentration was significantly increased in T-DPSC-CM, we interrogated whether Ang1 in T-DPSC-CM could activate its receptor Tie2 and the downstream signaling. ECs were treated with T-DPSC-CM or Ang1 for different time points. Western blot results showed that the expression of p-Tie2 significantly increased after treatment with T-DPSC-CM or Ang1, as well as VE-Cadherin (Fig. 6a, b, c). Taken together, TGF-β1 treatment induced DPSC secretion of Ang1 into CM, which in turn inhibited EC migration through activation of Tie2 and its downstream signaling.

### T-DPSCs enhanced vascular stability through Ang1/Tie2 and VEGF/VEGFR2 signaling

**Fig. 7:**
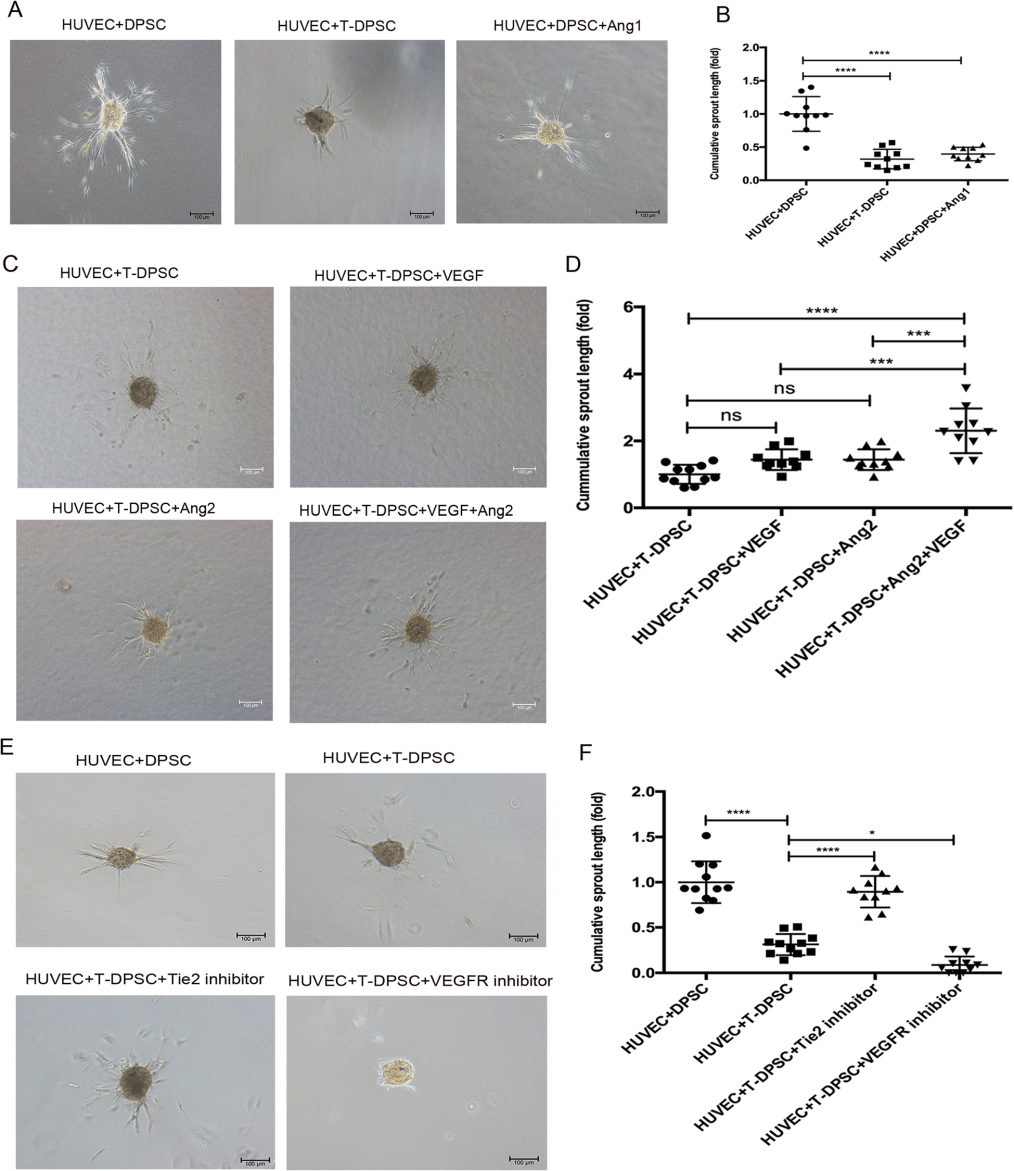
T-DPSCs inhibited HUVEC sprouting through Ang1/Tie2 and VEGF/VEGFR2 signaling. **a** T-DPSCs and exogenous Ang1 inhibited endothelial sprouting. Representative images of in vitro sprouting assay using HUVECs + DPSCs, HUVECs + T-DPSCs, and HUVECs + DPSCs + Ang1 co-culture spheroids, respectively. **b** Quantification of cumulative length of sprouting assay using ImageJ software. **c**, **d** Sprouting assay of HUVEC and T-DPSC co-culture spheroids stimulated with VEGF (20 ng/mL) and Ang2 (1000 ng/mL). Representative images (**c**) and quantification of cumulative length of sprouting (**d**). **e**, **f** HUVECs were pretreated with the Tie2 inhibitor (5 μM) or VEGFR2 inhibitor (5μM) for 12 h before co-cultured with T-DPSCs to form spheroids. Sprouting images (**e**) and quantification of cumulative length (**f**). Data are mean ± standard error for *n* = 3 replicates, *p <0.05, ***p <0.001, ****p <0.0001

To further investigate whether T-DPSCs maintain vessel stability through Ang1/Tie2 and VEGF/VEGFR2 signaling, in vitro 3D sprouting assay was performed. Co-culture spheroids of HUVECs and DPSCs, HUVECs and T-DPSCs, and HUVECs, DPSCs, and Ang1 were embedded in collagen gel and sprouting length was measured after 24 h. The sprouting assay showed that both T-DPSCs and exogenous Ang1 significantly inhibited EC sprouting in a 3D spheroidal co-culture model (Fig. 7a, b). In order to analyze the synergistic effects of VEGF and Ang2 on EC sprouting, the HUVEC and T-DPSC co-cultured spheroids were co-stimulated with VEGF and Ang2. The results showed that neither VEGF nor Ang2 alone can reverse the sprouting inhibition by T-DPSCs in co-culture spheroids, whereas co-stimulation with VEGF and Ang2 can significantly induce EC sprouting (Fig. 7c, d). When HUVECs were pretreated with a Tie2 inhibitor or VEGFR2 inhibitor, the Tie2 inhibitor reversed the sprouting inhibition by T-DPSCs (Fig. 7e, f). Conversely, the VEGFR2 inhibitor boosted the sprouting inhibition by T-DPSCs through blocking VEGF/VEGFR2 signaling activation (Fig. 7e, f).

**Fig. 8:**
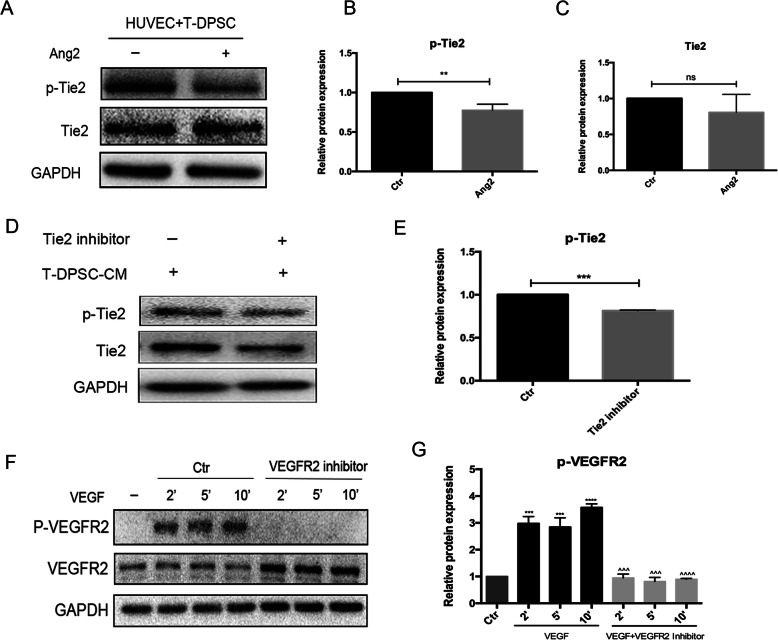
The expression of p-Tie2 and p-VEGFR2 in HUVECs. **a**–**c** Western blot analysis of the expression of p-Tie2 and Tie2 in HUVEC/T-DPSC co-culture with or without high concentration Ang2 (1000 ng/mL) for 24 h. **d**, **e** HUVECs were pretreated with the Tie2 inhibitor (5 μM) for 12h, then treated with T-DPSC-CM or Ang1 for 30 min. Western blot analysis of the expression of p-Tie2 in HUVECs. **f**, **g** HUVECs were pretreated with the VEGFR2 inhibitor (5 μM) for 12h, then treated with VEGF for 2, 5, and 10 min. Western blot analysis of the expression of p-VEGFR2 in HUVECs. Data are mean ± standard error for *n* = 3 replicates, *p <0.05, **p <0.01, ***p <0.001, ****p <0.0001

In order to explore the role of exogenous Ang2 on the expression of p-Tie2 and Tie2 in HUVEC/T-DPSC co-culture. The expression of p-Tie2 and Tie2 were detected by western blots in HUVEC/T-DPSC co-culture with or without high concentration of Ang2 (1000 ng/mL). The results showed Ang2 significantly suppressed Tie2 phosphorylation and had no effect on Tie2 expression (Fig. 8a–c). In addition, the efficiency of the Tie2 inhibitor and VEGFR2 inhibitor was confirmed by western blots. The expression of p-Tie2 (Fig. 8d, e) and p-VEGFR2 (Fig. 8f, g) significantly decreased after treatment with the Tie2 inhibitor or VEGFR2 inhibitor under the stimulation of T-DPSC-CM or VEGF.

## Discussion

Angiogenesis is a multi-step process that generates functional blood vessels, during which endothelial cells proliferate, migrate, and assemble to construct the interior surface of the vessels, and perivascular cells (SMCs and pericytes) are recruited to cover the exterior surface [27]. Initially, Ang2 disassociates the stabilized endothelial interactions with adjacent ECs and pericytes, and then, VEGF promotes EC proliferation, migration, and sprouting [28, 29]. In the later stage, PDGF released by ECs activates and recruits pericytes to the nascent vessels, while Ang1, which is released from pericytes, acts as a pro-maturation factor to promote EC and pericyte interactions and vessel maturation [10]. Through direct interaction with ECs, perivascular cells play a key role in maintaining the stability of mature blood vessels, which is of paramount importance for the vessels to carry out their physiological function [8].

TGF-β1 mediates several biological activities, such as tissue homeostasis [30], cell self-renewal and quiescence [31, 32], and cell differentiation [33]. In our previous study, we demonstrated that TGF-β1 induced human exfoliated deciduous teeth (SHED) differentiation into SMCs [5]. Furthermore, it has been demonstrated that TGF-β1 treatment induced DPSC differentiation into bladder SMCs [34]. DPSCs and SHED, which are derived from the pulp of teeth, possess the properties of MSCs and demonstrate the ability to differentiate into various primary cell phenotypes, such as neuronal, endothelial, and SMCs [5, 35]. Both DPSCs and SHED have been investigated for their angiogenic potentials in tissue vascularization, vasculature engineering, and treatment of ischemic diseases [6, 36, 37]. However, a relatively small volume of pulp tissue available within the exfoliated deciduous compared to that within the wisdom teeth makes it much difficult to isolate sufficient cells [38]. Therefore, in this project, DPSCs were utilized instead of SHED.

In the current study, we examined whether TGF-β1 treatment could switch DPSC phenotype into SMC-like cells, by which the newly formed vessels by HUVECs could be stabilized. Therefore, we first verified that TGF-β1 can drive DPSC differentiation into functional SMCs, as shown by high expression of α-SMA and SM22α, which are the most widely recognized markers of SMCs [39, 40]. Additionally, in vitro contraction assay that utilized the well-established collagen gel model was performed to assess the contractility of SMCs [26]. It was found that T-DPSCs have greater contractibility than DPSCs, indicating that T-DPSCs are functionally closer to SMCs.

In mammalians, five main ligands (VEGF-A, VEGF-B, VEGF-C, VEGF-D, and placenta-derived growth factor (PIGF)) and three receptors (VEGFR1, VEGFR2, and VEGFR3) have been found in the VEGF family [41]. VEGF-A, VEGF-B, and PIGF stimulate the growth of blood vessels through VEGFR1 and VEGFR2, while VEGF-C and VEGF-D regulate lymphatic angiogenesis through VEGFR3 [42]. VEGF-A, also often referred to as VEGF, is the best characterized and mostly utilized in VEGF/VEGFR2 signaling studies [43]. VEGF165 is the main isoform of VEGF-A and found to be expressed in many cell types [44]. VEGF exerts its biological role by interacting with cell surface receptors, such as VEGFR1 and VEGFR2, which are expressed on vascular endothelial cells [45]. In particular, VEGFR2 has been identified as the main receptor which is responsible for the regulation of the pro-angiogenic effects of VEGF-A [41, 46]. Therefore, VEGF-165 was utilized in this study to induce angiogenic sprouting.

In vitro 3D spheroidal sprouting assay has been used to assess interactions between ECs and various other cells, including MSCs, SMCs, pericytes, and tumor cells [47–50]. Using this model, the synergistic effect of VEGF and Ang2 in EC and SMC co-culture spheroids was demonstrated and the role of Tie2 in EC and pericyte co-culture spheroids was explored [47, 48]. Similarly, to assess the ability of T-DPSCs in stabilizing the HUVECs generated vessels, the in vitro 3D spheroidal sprouting assay was performed. The results showed that T-DPSCs significantly inhibited the EC sprouting compared to DPSCs. Confocal microscope images showed that T-DPSCs enclosed ECs in the sprouting structure, which suggested the T-DPSC function as pericyte-like cells in the process of angiogenesis sprouting.

Ang1, a pro-maturation factor, is responsible for blood vessel maturation through strengthening the interactions between perivascular cells and ECs [18]. It was well-known that Ang1/Tie2 signaling is the key contributor for maintaining the stability of the existing blood vessels [51]. Our results showed that TGF-β1 treatment activated the downstream signaling Smad2 and Smad3 via phosphorylation. The activated Smad2 and Smad3 associated with Smad4 are then translocated into the nucleus to trigger the target genes transcription, such as α-SMA, SM22α, Ang1, and VEGF [5, 52]. We assumed that T-DPSCs inhibited the endothelial sprouting in the 3D spheroidal co-culture model through Ang1/Tie2 signaling. In order to confirm our hypothesis, we first evaluated the dynamic changes of Ang1 and VEGF expression in DPSCs by western blot and ELISA after treatment with TGF-β1 from day 1 to 7. Both total and secreted Ang1 expression were significantly increased at day 5 after treatment with TGF-β1. The western blot results demonstrated that the peak of VEGF expression was at day 3 and then downregulated at days 5 and 7. The secreted VEGF assessed by ELISA had a similar trend, which was highest at day 3, and decreased at days 5 and 7. Consistently, it was reported that mesenchymal stem cells (MSCs), such as ASCs, following their differentiation into smooth muscle cells downregulated VEGF expression while upregulating α-SMA, SM22α expression [15, 16].

To further disclose the functional role of T-DPSCs, the CM of T-DPSCs was used to treat ECs. We found that T-DPSC-CM significantly inhibited EC migration, but had no effect on EC proliferation. The elevated level of Ang1 may be responsible for inhibiting EC migration. It was reported that Ang1 induces *trans*-association of Tie2 at endothelial cell-cell contacts. The *trans*-association of Tie2 further associates with vascular endothelial protein tyrosine phosphatase (VE-PTP) and enhances endothelial cell-cell adhesions [53]. The enhanced adhesion between ECs promotes their quiescent status, making the cell assembling more resistant to be broken by angiogenic factors such as VEGF [54]. On the other hand, the secreted VEGF in T-DPSC-CM was at the same level as that in DPSC-CM after treatment with TGF-β1 for 7 days. As the level of Ang1 was significantly elevated in T-DPSC-CM, the ratio of Ang1/VEGF was much higher in T-DPSC-CM than that in DPSC-CM, which in turn suppressed EC migration compared to DPSC-CM. Due to the same level of VEGF in T-DPSC-CM and DPSC-CM, this could explain why there was no difference on proliferation of ECs in both groups.

Besides the role of Ang1 in inhibiting EC migration, we found that Ang1 inhibited endothelial sprouting in 3D HUVEC and DPSC co-culture spheroidal model. Ang1 could inhibit endothelial sprouting by promoting the interactions between HUVECs and DPSCs via Ang1/Tie2 signaling [55]. Additionally, the cumulative sprouting length was significantly increased when HUVECs were pretreated with the Tie2 inhibitor, while the cumulative sprouting length was significantly decreased when HUVECs were pretreated with VEGFR2 inhibitor. The Tie2 inhibitor blocks Ang1 binding to its receptor Tie2, halting the accumulation and internalization of endothelial cadherin (VE-cadherin) through inhibiting the activation of Src [56]. Thus, it weakened the interactions between ECs and T-DPSCs and further increased the EC sprouting. On the contrary, the VEGFR2 inhibitor blocks VEGF binding to VEGFR2, inhibiting EC proliferation and migration through PI3K signaling and further decreased EC sprouting [57, 58]. These results indicated that Ang1/Tie2 and VEGF/VEGFR2 signaling both were involved in the angiogenesis sprouting of co-culture HUVECs and DPSCs. Ang2, as an Ang1 antagonist, plays an important role in Ang1-mediated vessel maturation [59]. Ang2, which is mainly produced by endothelial cells, destabilizes the interaction between ECs and perivascular cells by competing with Ang1 binding to Tie2 [60]. To further explore the function of Ang2 and the synergistic effect of Ang2 and VEGF in the process of sprouting, we performed in vitro sprouting assay co-stimulated with Ang2 and VEGF. The results showed that neither Ang2 nor VEGF individually was able to significantly induce EC sprouting. However, co-stimulation of HUVEC and T-DPSC co-culture spheroids with Ang2 and VEGF drastically increased EC sprouting. Ang2 was reported as a partial agonist/antagonist of Tie2 pathway that participates in the regulation of endothelium [61]. Similarly, our results demonstrated that a high concentration of Ang2 (1000 ng/mL) suppressed Tie2 phosphorylation in HUVEC and T-DPSC co-culture. Thus, this could explain why Ang2 and VEGF co-stimulation reversed the sprouting inhibition by T-DPSC. In consistent with our findings, a previous study showed that co-stimulation of EC and SMC co-culture spheroids with VEGF and Ang2 significantly induced sprouting in collagen gels, while neither VEGF nor Ang2 was able to induce sprouting [47]. Ang2 breaks down the binding of Ang1/Tie2 promoting perivascular cell detachment from the endothelium, and VEGF further activates EC migration to form new vessel sprouts [59, 62]. Therefore, these findings demonstrated that there is a facilitatory role between Ang2 and VEGF in the process of sprouting.

**Fig. 9:**
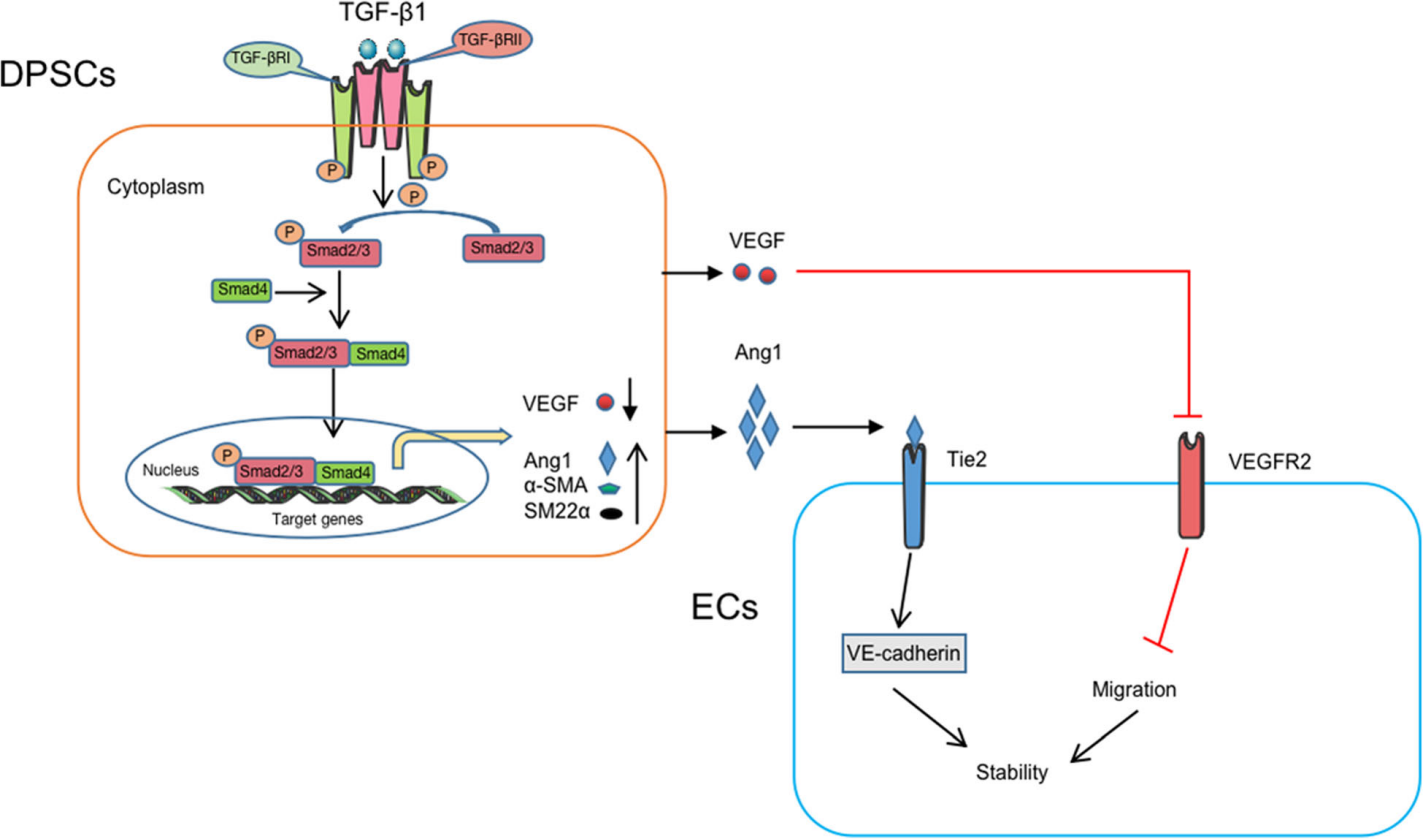
The proposed molecular mechanism on how TGF-β1 regulate the role of DPSCs in vascular stabilization. TGF-β1 treatment activated Smad2 and Smad3 via phosphorylation, and the activated Smad2 and Smad3 associated with Smad4 regulated Ang1 and VEGF expression besides α-SMA, SM22α. After secretion, Ang1 derived from DPSCs activated its receptor, Tie2, on the HUVEC membrane surface. The activated Ang1/Tie2 promoted VE-Cadherin expression, enhancing cell-cell adhesion and blood vessel stability. The secreted VEGF from DPSCs decreased gradually as DPSCs differentiated into pericyte-like cells, which in turn attenuated VEGF/VEGFR2 signaling, inhibiting HUVEC migration

In summary, we revealed that TGF-β1 treatment triggered Smad2 and Smad3 phosphorylation, and the activated Smad2 and Smad3 further induced Ang1 and VEGF gene and protein expression besides α-SMA and SM22α. After secretion, Ang1 derived from DPSCs activated its receptor, Tie2, on the membrane surface of HUVECs. The activated Ang1/Tie2 increased VE-Cadherin expression, which enhances cell-cell adhesion and blood vessel stability. The secreted VEGF from DPSCs decreased gradually as DPSCs differentiated into pericyte-like cells, which in turn attenuated VEGF/VEGFR2 signaling, inhibiting HUVEC migration. Thus, these results suggest the proposed molecular mechanism on how TGF-β1 regulates the role of DPSCs in vascular stabilization via Ang1/Tie2 and VEGF/VEGFR2 signaling (Fig. 9).

The results of this study showed that TGF-β1 promoted DPSC differentiation into functional pericyte-like cells which would expand the prospect of DPSCs’ translational applications. In fact, DPSCs, as a typical MSC type, have been investigated for osteogenesis, angiogenesis, neurogenesis, and immunomodulation [63, 64]. By regulating the associated signaling pathways as shown in this study, novel functions of DPSCs could be induced, which may facilitate the establishment of new therapeutic modalities in vascular tissue engineering.

## Conclusions

The current study demonstrated that DPSCs can be differentiated into functional SMCs by TGF-β1. Further, T-DPSCs can maintain the stability of vessels in a 3D co-culture spheroid model through Ang1/Tie2 and VEGF/VEGFR2 signaling.

## Supplementary Information

**Additional file 1.**

## Data Availability

The datasets used and/or analyzed during the current study are available from the corresponding author on reasonable request.

## Abbreviations

SMCs: Smooth muscle cells
DPSCs: Dental pulp cells
MSCs: Mesenchymal stem cells
T-DPSCs: TGF-β1-treated DPSCs for 7 days
HUVECs: Human umbilical vein endothelial cells
ECs: Endothelial cells
HBVPs: Human brain vascular pericytes
SHED: Stem cells from human exfoliated deciduous teeth
CM: Conditioned media
VEGF: Vascular endothelial growth factor
TGF-β1: Transforming growth factor beta 1
α-SMA: α-Smooth muscle actin
PDGFR: Platelet-derived growth factor receptors
PDGF: Platelet-derived growth factor
BMMSCs: Bone marrow mesenchymal stem cells
ASCs: Adipose stem cells
Ang1: Angiopoietin 1
Ang2: Angiopoietin 2
VE-PTP: Endothelial protein tyrosine phosphatase
VE-cadherin: Vascular endothelial cadherin
GAPDH: Glyceraldehyde 3-phosphate dehydrogenase

## References

[1] Sloan AJ, Waddington RJ (2009). Dental pulp stem cells: what, where, how?. Int. J. Paediatr. Dent..

[2] Ledesma-Martínez E, Mendoza-Núñez VM, Santiago-Osorio E (2016). Mesenchymal stem cells derived from dental pulp: a review. Stem Cells Int..

[3] Janebodin K, Zeng Y, Buranaphatthana W, Ieronimakis N, Reyes M (2013). VEGFR2-dependent angiogenic capacity of pericyte-like dental pulp stem cells. J Dent Res..

[4] Dissanayaka WL, Zhan X, Zhang C, Hargreaves KM, Jin L, Tong EH (2012). Coculture of dental pulp stem cells with endothelial cells enhances osteo-/odontogenic and angiogenic potential in vitro. J Endod..

[5] Xu JG, Zhu SY, Heng BC, Dissanayaka WL, Zhang CF (2017). TGF-β1-induced differentiation of SHED into functional smooth muscle cells. Stem Cell Res Ther..

[6] Gong T, Xu J, Heng B, Qiu S, Yi B, Han Y, Lo ECM, Zhang C (2019). EphrinB2/EphB4 signaling regulates DPSCs to induce sprouting angiogenesis of endothelial cells. J Dent Res..

[7] Oh M, Zhang Z, Mantesso A, Oklejas A, Nör J (2020). Endothelial-initiated crosstalk regulates dental pulp stem cell self-renewal. J Dent Res..

[8] Carmeliet P (2000). Mechanisms of angiogenesis and arteriogenesis. Nat Med..

[9] Darland DC, D’Amore PA (1999). Blood vessel maturation: vascular development comes of age. J. Clin. Invest..

[10] Brudno Y, Ennett-Shepard AB, Chen RR, Aizenberg M, Mooney DJ (2013). Enhancing microvascular formation and vessel maturation through temporal control over multiple pro-angiogenic and pro-maturation factors. Biomaterials..

[11] Dissanayaka WL, Hargreaves KM, Jin L, Samaranayake LP, Zhang C (2015). The interplay of dental pulp stem cells and endothelial cells in an injectable peptide hydrogel on angiogenesis and pulp regeneration in vivo. Tissue Eng Part A..

[12] Yuan C, Wang P, Zhu L, Dissanayaka WL, Green DW, Tong EH (2015). Coculture of stem cells from apical papilla and human umbilical vein endothelial cell under hypoxia increases the formation of three-dimensional vessel-like structures in vitro. Tissue Eng Part A..

[13] Duffy GP, Ahsan T, O'brien T, Barry F, Nerem RM (2009). Bone marrow–derived mesenchymal stem cells promote angiogenic processes in a time-and dose-dependent manner in vitro. Tissue Eng Part A..

[14] Verseijden F, Posthumus-van Sluijs SJ, Pavljasevic P, Hofer SO, van Osch GJ, Farrell E (2010). Adult human bone marrow and adipose tissue derived stromal cells support the formation of prevascular-like structures from endothelial cells in vitro. Tissue Eng Part A..

[15] Merfeld-Clauss S, Lupov IP, Lu H, Feng D, Compton-Craig P, March KL, Traktuev DO (2014). Adipose stromal cells differentiate along a smooth muscle lineage pathway upon endothelial cell contact via induction of activin A. Circ Res..

[16] Merfeld-Clauss S, Lease BR, Lu H, March KL, Traktuev DO (2017). Adipose stromal cells differentiation toward smooth muscle cell phenotype diminishes their vasculogenic activity due to induction of activin A secretion. J Tissue Eng Regen Med..

[17] Yancopoulos GD, Davis S, Gale NW, Rudge JS, Wiegand SJ, Holash J (2000). Vascular-specific growth factors and blood vessel formation. Nature..

[18] Davis S, Aldrich TH, Jones PF, Acheson A, Compton DL, Jain V, Ryan TE, Bruno J, Radziejewski C, Maisonpierre PC, Yancopoulos GD (1996). Isolation of angiopoietin-1, a ligand for the TIE2 receptor, by secretion-trap expression cloning. Cell..

[19] Papapetropoulos A, Fulton D, Mahboubi K, Kalb RG, O'Connor DS, Li F, Altieri DC, Sessa WC (2000). Angiopoietin-1 inhibits endothelial cell apoptosis via the Akt/survivin pathway. J Biol Chem..

[20] Kim I, Kim HG, So J-N, Kim JH, Kwak HJ, Koh GY (2000). Angiopoietin-1 regulates endothelial cell survival through the phosphatidylinositol 3′-kinase/Akt signal transduction pathway. Circ Res..

[21] Thomas M, Augustin HG (2009). The role of the angiopoietins in vascular morphogenesis. Angiogenesis..

[22] Augustin HG, Koh GY, Thurston G, Alitalo K (2009). Control of vascular morphogenesis and homeostasis through the angiopoietin–Tie system. Nat Rev Mol Cell Biol..

[23] Kurpinski K, Lam H, Chu J, Wang A, Kim A, Tsay E, Agrawal S, Schaffer DV, Li S (2010). Transforming growth factor-β and notch signaling mediate stem cell differentiation into smooth muscle cells. Stem Cells..

[24] Shi N, Xie W-B, Chen S-Y (2012). Cell division cycle 7 is a novel regulator of transforming growth factor-β-induced smooth muscle cell differentiation. J Biol Chem..

[25] Shah S, Lee H, Park YH, Jeon E, Chung HK, Lee ES (2019). Three-dimensional angiogenesis assay system using co-culture spheroids formed by endothelial colony forming cells and mesenchymal stem cells. J Vis Exp..

[26] Ngo P, Ramalingam P, Phillips JA, Furuta GT (2006). Collagen gel contraction assay. Methods Mol Biol..

[27] Adams RH, Alitalo K (2007). Molecular regulation of angiogenesis and lymphangiogenesis. Nat Rev Mol Cell Biol..

[28] Roviezzo F, Tsigkos S, Kotanidou A, Bucci M, Brancaleone V, Cirino G, Papapetropoulos A (2005). Angiopoietin-2 causes inflammation in vivo by promoting vascular leakage. J Pharmacol Exp Ther..

[29] Scharpfenecker M, Fiedler U, Reiss Y, Augustin HG (2005). The Tie-2 ligand angiopoietin-2 destabilizes quiescent endothelium through an internal autocrine loop mechanism. J Cell Sci..

[30] Annes JP, Munger JS, Rifkin DB (2003). Making sense of latent TGFβ activation. J Cell Sci..

[31] Wilson A, Laurenti E, Oser G, van der Wath RC, Blanco-Bose W, Jaworski M, Offner S, Dunant CF, Eshkind L, Bockamp E, Lió P, MacDonald HR, Trumpp A (2008). Hematopoietic stem cells reversibly switch from dormancy to self-renewal during homeostasis and repair. Cell..

[32] Blank U, Karlsson G, Moody JL, Utsugisawa T, Magnusson M, Singbrant S, Larsson J, Karlsson S (2006). Smad7 promotes self-renewal of hematopoietic stem cells. Blood..

[33] Derynck R, Zhang YE (2003). Smad-dependent and Smad-independent pathways in TGF-β family signalling. Nature..

[34] Song B, Jiang W, Alraies A, Liu Q, Gudla V, Oni J (2016). Bladder smooth muscle cells differentiation from dental pulp stem cells: future potential for bladder tissue engineering. Stem Cells Int..

[35] Perry BC, Zhou D, Wu X, Yang F-C, Byers MA, Chu T-MG, Hockema JJ, Woods EJ, Goebel WS (2008). Collection, cryopreservation, and characterization of human dental pulp–derived mesenchymal stem cells for banking and clinical use. Tissue Engineering Part C: Methods..

[36] Lan X, Sun Z, Chu C, Boltze J, Li S (2019). Dental pulp stem cells: an attractive alternative for cell therapy in ischemic stroke. Front. Neurol..

[37] Guo H, Zhao W, Liu A, Wu M, Shuai Y, Li B, Huang X, Liu X, Yang X, Guo X, Xuan K, Jin Y (2020). SHED promote angiogenesis in stem cell-mediated dental pulp regeneration. Biochem. Biophys. Res. Commun..

[38] Kawashima N, Noda S, Yamamoto M, Okiji T (2017). Properties of dental pulp–derived mesenchymal stem cells and the effects of culture conditions. J Endod..

[39] Owens GK, Kumar MS, Wamhoff BR (2004). Molecular regulation of vascular smooth muscle cell differentiation in development and disease. Physiol Rev..

[40] Rensen S, Doevendans P, Van Eys G (2007). Regulation and characteristics of vascular smooth muscle cell phenotypic diversity. Neth Heart J..

[41] Ferrara N, Gerber H-P, LeCouter J (2003). The biology of VEGF and its receptors. Nat Med..

[42] Roberts E, Cossigny DA, Quan GM (2013). The role of vascular endothelial growth factor in metastatic prostate cancer to the skeleton. Prostate Cancer..

[43] Shibuya M (2011). Vascular endothelial growth factor (VEGF) and its receptor (VEGFR) signaling in angiogenesis: a crucial target for anti-and pro-angiogenic therapies. Genes Cancer..

[44] Ancelin M, Buteau-Lozano H, Meduri G, Osborne-Pellegrin M, Sordello S, Plouët J (2002). A dynamic shift of VEGF isoforms with a transient and selective progesterone-induced expression of VEGF189 regulates angiogenesis and vascular permeability in human uterus. Proc Natl Acad Sci U S A..

[45] Dvorak HF (2002). Vascular permeability factor/vascular endothelial growth factor: a critical cytokine in tumor angiogenesis and a potential target for diagnosis and therapy. J. Clin. Oncol..

[46] Cross MJ, Dixelius J, Matsumoto T, Claesson-Welsh L (2003). VEGF-receptor signal transduction. Trends Biochem. Sci..

[47] Korff T, Kimmina S, MARTINY-BARON G, Augustin HG (2001). Blood vessel maturation in a 3-dimensional spheroidal coculture model: direct contact with smooth muscle cells regulates endothelial cell quiescence and abrogates VEGF responsiveness. FASEB J..

[48] Teichert M, Milde L, Holm A, Stanicek L, Gengenbacher N, Savant S (2017). Pericyte-expressed Tie2 controls angiogenesis and vessel maturation. Nat Commun..

[49] Vorwald CE, Joshee S, Leach JK. Spatial localization of endothelial cells in heterotypic spheroids influences Notch signaling. J Mol Med (Berl). 2020;98:425–35. 10.1007/s00109-020-01883-1 PMC7083693 32020237

[50] Shoval H, Karsch-Bluman A, Brill-Karniely Y, Stern T, Zamir G, Hubert A (2017). Tumor cells and their crosstalk with endothelial cells in 3D spheroids. Sci Rep..

[51] Fukuhara S, Sako K, Noda K, Zhang J, Minami M, Mochizuki N (2010). Angiopoietin-1/Tie2 receptor signaling in vascular quiescence and angiogenesis. Histol Histopathol..

[52] Ferrari G, Cook BD, Terushkin V, Pintucci G, Mignatti P (2009). Transforming growth factor-beta 1 (TGF-β1) induces angiogenesis through vascular endothelial growth factor (VEGF)-mediated apoptosis. J. Cell. Physiol..

[53] Saharinen P, Eklund L, Miettinen J, Wirkkala R, Anisimov A, Winderlich M, Nottebaum A, Vestweber D, Deutsch U, Koh GY, Olsen BR, Alitalo K (2008). Angiopoietins assemble distinct Tie2 signalling complexes in endothelial cell–cell and cell–matrix contacts. Nat Cell Biol..

[54] Carmeliet P, Jain RK (2011). Molecular mechanisms and clinical applications of angiogenesis. Nature..

[55] Gu A, Shively JE (2011). Angiopoietins-1 and-2 play opposing roles in endothelial sprouting of embryoid bodies in 3D culture and their receptor Tie-2 associates with the cell–cell adhesion molecule PECAM1. Exp Cell Res..

[56] Gavard J, Patel V, Gutkind JS (2008). Angiopoietin-1 prevents VEGF-induced endothelial permeability by sequestering Src through mDia. Dev Cell..

[57] Holmqvist K, Cross MJ, Rolny C, Hägerkvist R, Rahimi N, Matsumoto T, Claesson-Welsh L, Welsh M (2004). The adaptor protein shb binds to tyrosine 1175 in vascular endothelial growth factor (VEGF) receptor-2 and regulates VEGF-dependent cellular migration. J Biol Chem..

[58] Dayanir V, Meyer RD, Lashkari K, Rahimi N (2001). Identification of tyrosine residues in vascular endothelial growth factor receptor-2/FLK-1 involved in activation of phosphatidylinositol 3-kinase and cell proliferation. J Biol Chem..

[59] Maisonpierre PC, Suri C, Jones PF, Bartunkova S, Wiegand SJ, Radziejewski C, Compton D, McClain J, Aldrich TH, Papadopoulos N, Daly TJ, Davis S, Sato TN, Yancopoulos GD (1997). Angiopoietin-2, a natural antagonist for Tie2 that disrupts in vivo angiogenesis. Science..

[60] Stratmann A, Risau W, Plate KH (1998). Cell type-specific expression of angiopoietin-1 and angiopoietin-2 suggests a role in glioblastoma angiogenesis. Am J Pathol..

[61] Yuan HT, Khankin EV, Karumanchi SA, Parikh SM (2009). Angiopoietin 2 is a partial agonist/antagonist of Tie2 signaling in the endothelium. Mol. Cell. Biol..

[62] Ferrara N (2000). VEGF: an update on biological and therapeutic aspects. Curr Opin Biotechnol..

[63] Nakashima M, Iohara K, Murakami M (2013). Dental pulp stem cells and regeneration. Endodontic Topics..

[64] Zayed M, Iohara K (2020). Immunomodulation and regeneration properties of dental pulp stem cells: a potential therapy to treat coronavirus disease 2019. Cell Transplant..

